# mGlu5 receptors and cellular prion protein mediate amyloid-β-facilitated synaptic long-term depression *in vivo*

**DOI:** 10.1038/ncomms4374

**Published:** 2014-03-04

**Authors:** Neng-Wei Hu, Andrew J. Nicoll, Dainan Zhang, Alexandra J. Mably, Tiernan O’Malley, Silvia A. Purro, Cassandra Terry, John Collinge, Dominic M. Walsh, Michael J. Rowan

**Affiliations:** 1 Department of Pharmacology and Therapeutics, and Trinity College Institute of Neuroscience, Biotechnology Building, Trinity College Dublin, Dublin 2, Ireland; 2 Medical Research Council Prion Unit and Department of Neurodegenerative Disease, UCL Institute of Neurology, Queen Square, London WC1N 3BG, UK; 3 Laboratory for Neurodegenerative Research, Center for Neurologic Diseases, Brigham & Women’s Hospital, Harvard Institute of Medicine, 77 Avenue Louis Pasteur, Boston, Massachusetts 02115, USA

## Abstract

NMDA-type glutamate receptors (NMDARs) are currently regarded as paramount in the potent and selective disruption of synaptic plasticity by Alzheimer’s disease amyloid β-protein (Aβ). Non-NMDAR mechanisms remain relatively unexplored. Here we describe how Aβ facilitates NMDAR-independent long-term depression of synaptic transmission in the hippocampus *in vivo*. Synthetic Aβ and Aβ in soluble extracts of Alzheimer’s disease brain usurp endogenous acetylcholine muscarinic receptor-dependent long-term depression, to enable long-term depression that required metabotropic glutamate-5 receptors (mGlu5Rs). We also find that mGlu5Rs are essential for Aβ-mediated inhibition of NMDAR-dependent long-term potentiation *in vivo*. Blocking Aβ binding to cellular prion protein with antibodies prevents the facilitation of long-term depression. Our findings uncover an overarching role for Aβ-PrP^C^-mGlu5R interplay in mediating both LTD facilitation and LTP inhibition, encompassing NMDAR-mediated processes that were previously considered primary.

## Abstract (web summary)

In Alzheimer's disease, the soluble amyloid beta peptide is known to modulate synaptic function by forming a complex with prion proteins and metabotropic glutamate receptors. Here, Hu *et al.* show that amyloid beta signalling via this complex facilitates the induction of long-term depression at synapses.

## Graphical abstract

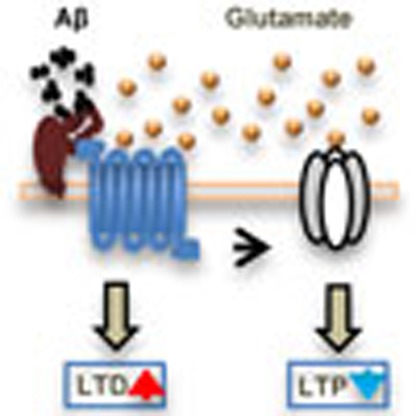

Increasing our understanding of how amyloid-β protein (Aβ) causes synaptic dysfunction should provide new means of therapeutically targeting early Alzheimer’s disease (AD)^1^. It is now well established that Aβ has rapid, profound and selective disruptive effects on synaptic plasticity of excitatory synaptic transmission in vulnerable brain regions, including the hippocampus^2^. In addition to causing strong inhibition of long-term potentiation (LTP), Aβ has been reported to enhance long-term depression (LTD). Most research has focused on the actions of Aβ on forms of LTP and LTD that require NMDA-type glutamate receptors (NMDARs)^3^,^4^,^5^,^6^. Indeed, as NMDAR-dependent LTP is likely to underlie synaptic memory mechanisms^7^, the inhibition of this form of LTP by Aβ is highly congruent with the ability of Aβ to impair learning and memory^8^,^9^. Somewhat similarly, excessive enhancement of LTD that requires NMDARs can cause memory retrieval deficits^10^,^11^. Remarkably, the disruption of NMDAR-dependent synaptic plasticity by Aβ is itself mediated through NMDARs, in particular, those containing the GluN2B subunit^12^,^13^,^14^,^15^.

In contrast, little is known about how Aβ affects forms of synaptic plasticity that do not require NMDARs. Whereas Aβ potently inhibits acetylcholine-induced LTP^16^, NMDAR-independent LTP induced by strong high-frequency conditioning stimulation (HFS) appears to be resistant to disturbance by Aβ^17^ in the hippocampus *in vitro*. Recently, Aβ was reported to enable an NMDAR-independent LTD that was blocked by metabotropic glutamate-5 receptor (mGlu5R) antagonists in hippocampal slices^5^,^8^. Indeed, synaptically evoked activation of mGlu5R or other similar G-protein coupled receptors including M1 muscarinic acetylcholine receptors (mAChRs) can induce LTD that does not require NMDARs^11^,^18^,^19^,^20^. Moreover, mAChR-dependent LTD has been proposed to underlie visual recognition memory in the perirhinal cortex^21^ and to provide a neurophysiological basis for preserved memory function in the ageing hippocampus^22^. Considering the early vulnerability of cholinergic pathways and related signalling in AD^23^,^24^, we hypothesize that Aβ would inhibit mAChR-dependent LTD.

Remarkably, *in vivo* exposure to low-dose Aβ facilitated an NMDAR-independent form of LTD but does not appear to affect mAChR-dependent LTD. This Aβ-facilitated LTD is found to be mGlu5R-dependent. Moreover, Aβ-mediated inhibition of LTP is also dependent on metabotropic glutamate-5 receptors (mGlu5Rs), indicating a key overarching role of this glutamate receptor subtype. We also discover that cellular PrP, a receptor for certain synaptotoxic Aβ assemblies^25^,^26^, is necessary for Aβ to facilitate LTD. These data are strongly congruent with recent molecular evidence that Aβ and cellular prion protein (PrP^C^) form a complex with mGlu5R at the postsynaptic density^27^ and thereby disrupt synaptic plasticity.

## Results

### *In vivo* induction of mAChR-dependent LTD

**Figure 1:**
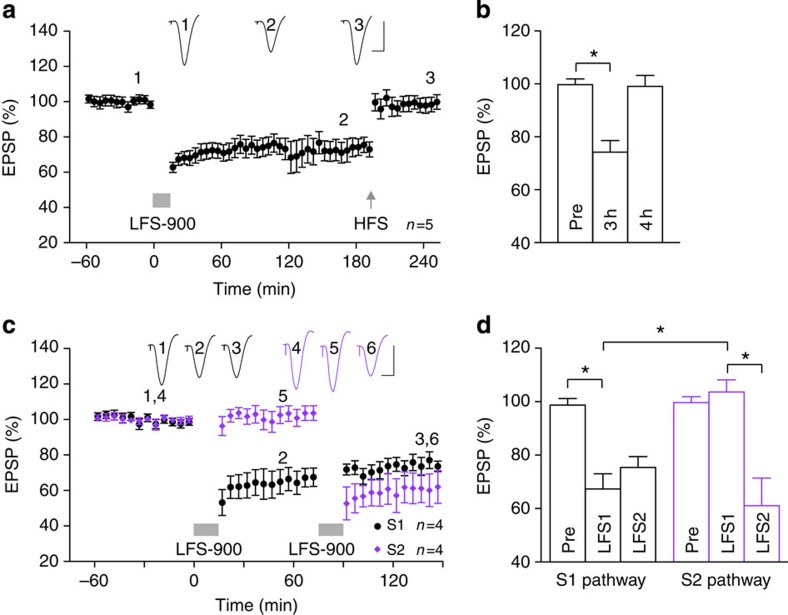
Low-frequency stimulation induces input-selective and reversible long-term depression at CA3-to-CA1 synapses *in vivo.* (**a**,**b**) Application of strong LFS (horizontal bar, LFS-900; 900 pulses at 1 Hz) induced robust and stable LTD. Three hours after LTD induction, application of high-frequency stimulation (arrow, HFS; 200 Hz) induced potentiation of synaptic transmission such that LTD was completely reversed. As summarized in (**b**), the EPSP decreased to 74.2±3.9%, at 3 h post LFS (3 h), *n*=5, *P*<0.05 compared with pre-LFS baseline (Pre); one-way ANOVA-Tukey. At 1 h post HFS (4 h) the EPSP reverted to 99.1±3.7%, *n*=5, *P*>0.05 compared with Pre; *P*<0.05 compared with LFS). (**c**,**d**) In four animals, two stimulation electrodes (S1, black and S2, purple) were implanted in different locations in the stratum radiatum to allow independent activation of the Schaffer collateral-commissural pathway. One hour after stable baseline recording from both S1 and S2 pathways, application of LFS-900 to S1 induced LTD in the S1 pathway but not in the S2 pathway. Conversely, 1 h after the first LFS, a second LFS was applied to S2 pathway that only induced LTD in pathway S2. As summarized in (**d**) One hour after application of LFS1, the EPSP in pathway S1 decreased to 67.4±5.6% (*P*<0.05 compared with Pre; one-way ANOVA-Tukey) but did not change significantly in the S2 pathway (103.6±4.5%, *P*>0.05 compared with Pre; *P*<0.05 compared with S1 pathway; *t*-test). In contrast, 1 h after application of LFS2, the EPSP was significantly reduced in the S2 pathway (61.1±10.4%, *P*<0.05 compared with Pre) but no further change was seen in the S1 pathway (75.4±4.1%, *P*>0.05 compared with EPSP amplitude pre-LFS2). Values are expressed as % mean baseline EPSP amplitude±s.e.m. Insets show representative EPSP traces at the times indicated. Calibration bars: vertical, 2 mV; horizontal, 10 ms. *P<0.05.

In order to study the effects of Aβ on mAChR-dependent LTD *in vivo*, we developed a novel induction protocol that makes use of the reported requirement for high-intensity pulses to ensure robust synaptic ACh release during low-frequency conditioning stimulation (LFS) in the neocortex^28^. We found that application of strong LFS, consisting of 900 high-intensity pulses at 1 Hz (LFS-900), in the stratum radiatum of anaesthetized rats triggered synaptic LTD that (i) was stable for ~3 h (Fig. 1a,b), (ii) was readily reversible by HFS (Fig. 1a,b) and (iii) was input specific (Fig. 1c,d).

**Figure 2:**
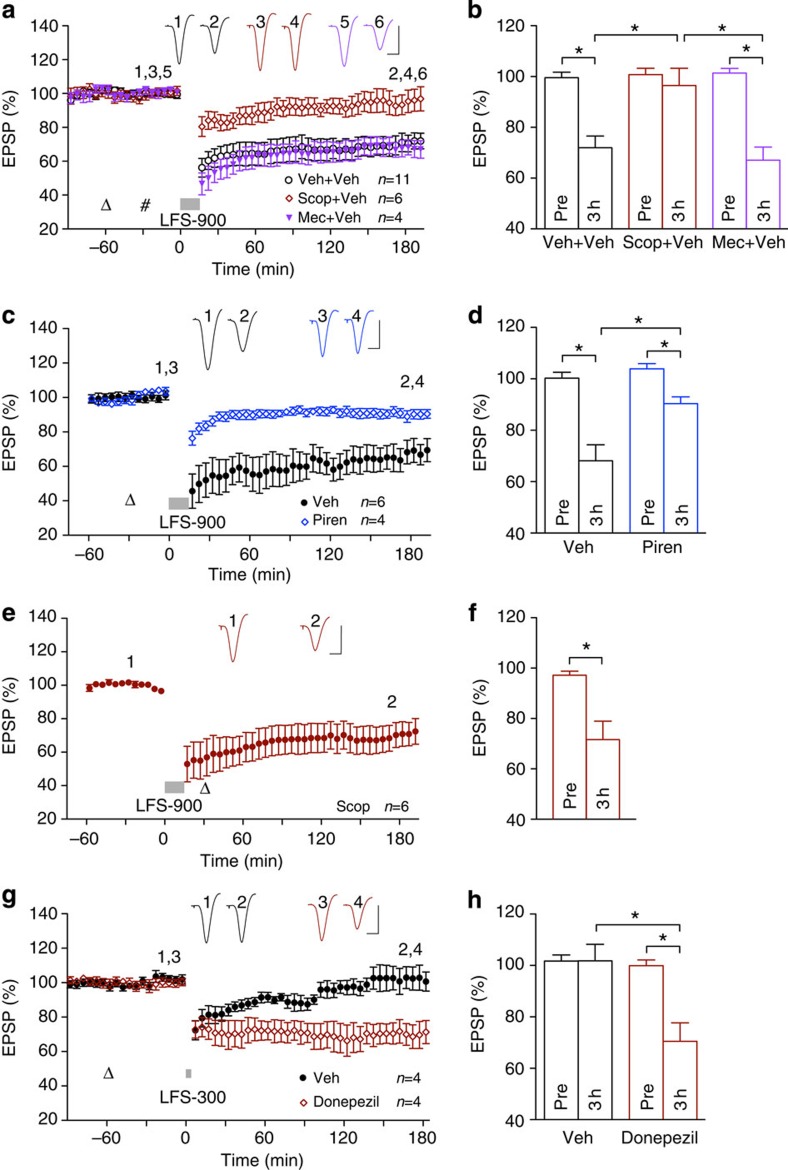
Muscarinic receptor-dependence of LTD *in vivo.* (**a**,**b**) Systemic injection of scopolamine (0.2 mg kg^−1^, i.p.), a muscarinic acetylcholine receptor antagonist, completely prevented LFS-induced LTD, whereas application of the nicotinic acetylcholine receptor antagonist mecamylamine (5 mg kg^−1^, i.p.) did not affect LTD induction. Open triangle, i.p.; hash, intracerebroventricular (i.c.v.). As summarized in (**b**) the EPSP decreased significantly to 72.0±4.4% in the vehicle control group and the mecamylamine group (67.1±4.9%, *n*=4, *P*<0.05 compared with Pre, *P*>0.05 compared with vehicle) but not in the scopolamine group (96.5±6.4%, *n*=6, *P*>0.05 compared with Pre, *P*<0.05 compared with vehicle); paired *t* and one-way ANOVA-Tukey. (**c**,**d**) LFS-900-induced LTD was also significantly reduced by treatment with the M1-selective mAChR antagonist pirenzepine (triangle, 50 nmol in 5 μl). As summarized in (**d**), the EPSP decreased to 67.5±4.5% and 90.4±2.1%, *n*=4, in vehicle- and pirenzepine-injected animals, respectively (*P*<0.05 compared with Pre and between groups; *t*-test). (**e**,**f**) Application of LFS-900 before the injection of scopolamine (triangle, 0.2 mg kg^−1^, i.p.) induced robust LTD (71.7±7.2%, *n*=6, *P*<0.05 compared with Pre; paired *t*). (**g**,**h**) The acetylcholinesterase inhibitor donepezil lowered the threshold to induce LTD. (**g**) The application of weak LFS (bar, LFS-300; 300 high-intensity pulses at 1 Hz) induced a transient synaptic depression in vehicle-injected animals (triangle), whereas the same protocol triggered a robust and stable LTD after acute injection of donepezil (1 mg kg^−1^, subcutaneously). (**h**) Veh: 101.8±6.3%; donepezil: 70.5±7.1% at 3 h after LFS. *P<0.05, *t*-test, *n*=4 per group. Values are mean±s.e.m. Calibration bars: vertical, 2 mV; horizontal, 10 ms.

Consistent with the essential requirement for activation of cholinergic mechanisms in the induction of this form of LTD, LFS-900 failed to induce LTD of synaptic transmission after pretreatment with the mAChR antagonist scopolamine (Fig. 2a,b). In contrast, the LTD was not dependent on the activation of nicotinic AChRs, the magnitude of LTD being unaffected by injection of the nicotinic AChR antagonist mecamylamine before LFS-900 (Fig. 2a,b). Consistent with a role for the M1 subtype of mAChR in LTD induction^19^, the M1-selective antagonist pirenzepine significantly reduced the magnitude of LTD (Fig. 2c,d). mAChR activation did not appear to be required for LTD maintenance/expression, as injection of scopolamine after the conditioning stimulation, using the same dose that completely prevented LTD induction, did not significantly affect the magnitude of LTD (Fig. 2e,f). Further evidence that physiological release of ACh is a key factor in LTD induction *in vivo* was the ability of an agent that enhances the effects of endogenously released ACh, the acetylcholinesterase inhibitor donepezil, to lower the threshold of LTD induction. Thus, we found that a relatively weak LFS conditioning protocol, consisting of 300 high-intensity pulses at 1 Hz (LFS-300) that was at or just below the threshold to induce significant LTD in vehicle-pretreated animals, triggered a large and robust LTD that was stable for at least 3 h in animals pretreated with donepezil (Fig. 2g,h). Moreover, as described below, the induction of this *in vivo* synaptically evoked mAChR-dependent LTD did not require the activation of NMDA or mGlu5Rs.

Because Aβ can interfere with mAChR-related signalling^29^, we went on to examine the ability of Aβ to disrupt this form of LTD.

### Aβ enhances an mAChR-independent form of LTD

**Figure 3:**
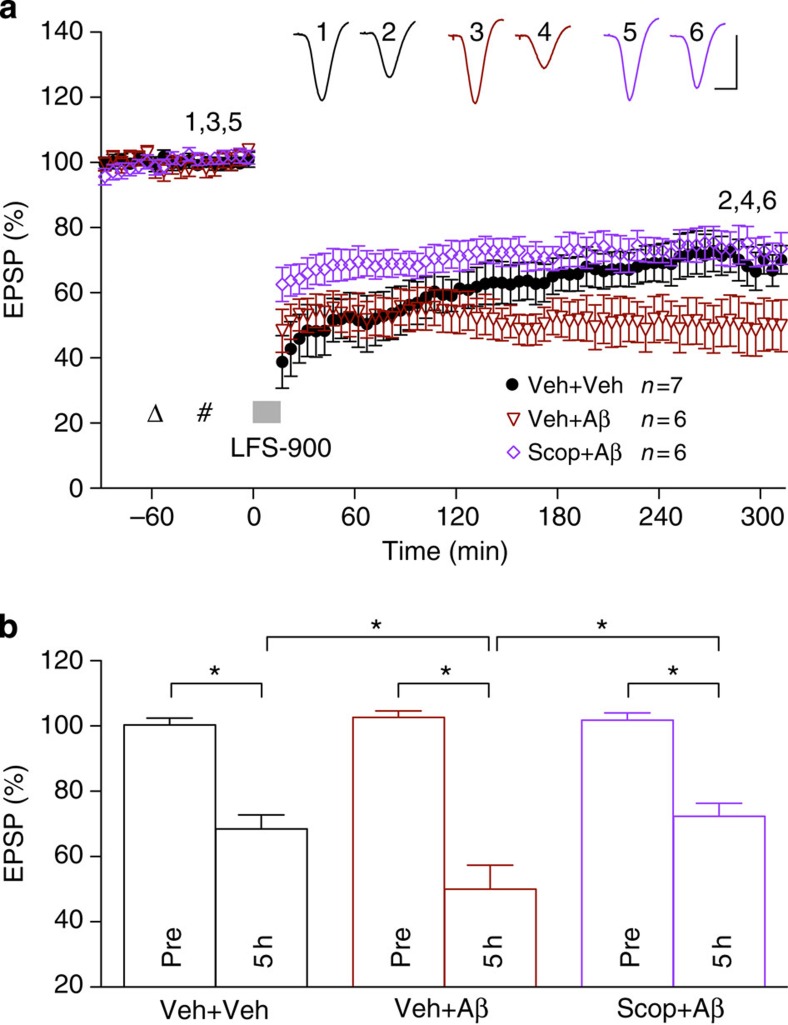
Intracerebroventricular injection of Aβ enables an additional LTD that is muscarinic receptor-independent. (**a**) Intracerebroventricular (hash) injection of 160 pmol soluble Aβ_1–42_ (5 μl of a 32-μM solution) 30 min before the application of LFS-900 did not affect the early phase (<2 h post LFS) but facilitated the late phase (3–5 h post LFS) of LFS-induced LTD. Systemic administration of scopolamine with the dose (open triangle; 0.2 mg kg^−1^, i.p.) that completely prevented LFS-induced LTD (see Fig. 2a,b) partly attenuated LFS-induced LTD in Aβ-treated animals. As summarized in (**b**), LFS-900 induced LTD measuring 68.5±4.3% in controls (*n*=7, *P*<0.05 compared with Pre), 50.0±7.4% in Aβ-pretreated rats (*n*=6, *P*<0.05 compared with Pre, *P*<0.05 compared with vehicle) and 72.3±4.0% in the scopolamine+Aβ group (*n*=6, *P*<0.05 compared with Pre, *P*<0.05 compared with the Aβ-treated group); paired *t* and one-way ANOVA-Tukey. Values are mean±s.e.m. Calibration: vertical, 2 mV; horizontal, 10 ms.

We investigated the effects of Aβ on synaptically evoked mAChR-dependent LTD *in vivo* by the injection of Aβ into the lateral cerebral ventricle via a cannula. Initially, we used a soluble synthetic Aβ_1–42_ preparation that had been centrifuged to remove any fibril aggregates. We chose a dose (160 pmol) of soluble Aβ_1–42_ that did not affect baseline synaptic transmission but strongly inhibited NMDAR-dependent LTP, as described below and previously^30^. To our surprise, in animals pre-injected with soluble Aβ_1–42_ the application of LFS-900 triggered an LTD that was more stable than the control LTD induced in the absence of Aβ. Thus, LTD induced in the presence of Aβ was stable during the 5-h recording period, whereas control LTD decayed significantly between 3 and 5 h post LFS (Fig. 3a,b). Although we had hypothesized that mAChR-dependent LTD would be inhibited by Aβ, we wondered whether this Aβ-facilitated LTD required mAChRs. We therefore pretreated the rats with scopolamine before Aβ. In contrast to control LTD, which was completely abrogated by the mAChR antagonist (Fig. 2a,b), the time course and magnitude of LTD was only partly reduced by scopolamine in Aβ-treated animals (Fig. 3a,b). These findings indicate that Aβ had enabled an additional LTD that was more stable and independent of mAChRs while at the same time leaving a residual mAChR-dependent LTD relatively unscathed.

**Figure 4:**
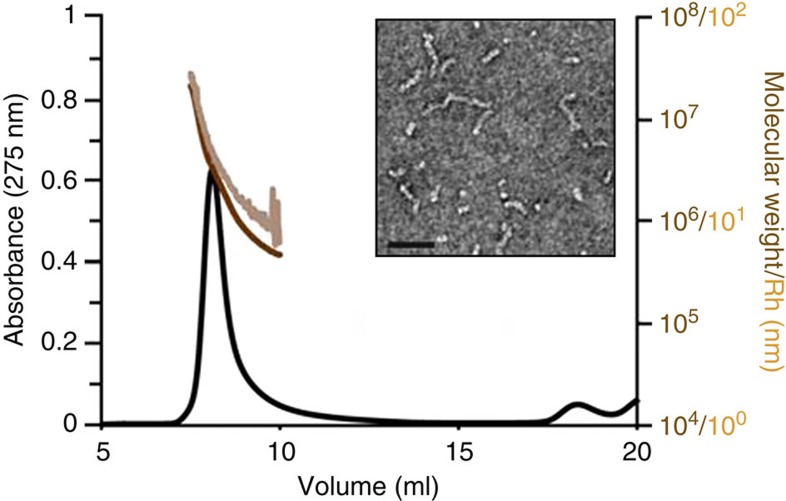
Characterization of protofibril Aβ_1–42_ preparation. Characterization of Aβ_1–42_ protofibrils by electron microscopy (EM), SEC and quasi-elastic light scattering (QELS) confirm these preparations contain predominantly protofibrillar assemblies of 10–100 nm in length with molecular weights of 10^5^–10^7^ and hydrodynamic radii of 8–50 nm. Scale bar, 50 nm.

**Figure 5:**
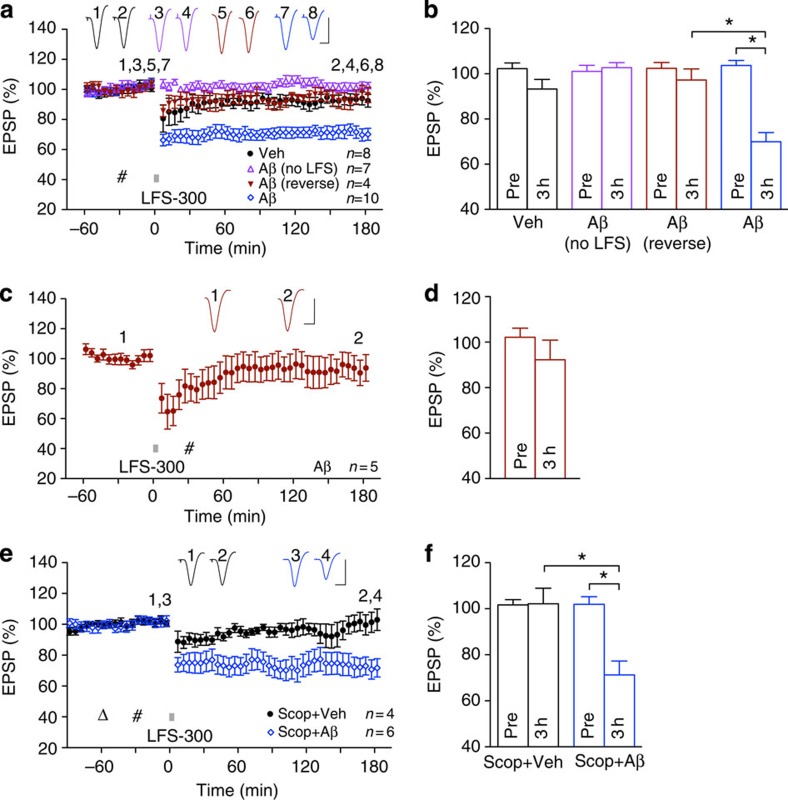
Aβ facilitates the induction of muscarinic receptor-independent LTD by weak low-frequency stimulation. (**a**,**b**) The application of weak LFS (bar, LFS-300; 300 high-intensity pulses at 1 Hz) triggered a robust and stable LTD after acute i.c.v. injection (hash) of 160 pmol Aβ_1–42_ but not vehicle or the reverse peptide Aβ_42–1_ (Aβ reverse). This dose of Aβ_1–42_ did not affect baseline synaptic transmission in the absence of LFS-300 (Aβ no LFS). Data for soluble and protofibril Aβ are combined and some animals had an additional separate i.c.v. injection of 5 μl vehicle 15 min before Aβ. As summarized in (**b**) at 3 h the EPSP measured 93.3±3.6% in controls (*n*=8, *P*>0.05 compared with Pre; paired *t*), 69.9±3.8% in Aβ-injected rats (*n*=10, *P*<0.05 compared with Pre and vehicle group; paired *t* and one-way ANOVA-Tukey) and 97.3±4.3% in reverse peptide (*n*=4, *P*>0.05 compared with Pre). Injection of Aβ_1–42_ (160 pmol, i.c.v.) did not affect baseline synaptic transmission (102.6±1.6% at 3 h, *n*=7, *P*>0.05 compared with Pre). (**c**,**d**) Aβ_1–42_, when administered 15 min after LFS-300 did not facilitate LTD. As summarized in (**d**) the EPSP was not significantly decreased at 3 h post LFS-300 (92.2 ±8.3%, *n*=5, *P*>0.05 compared with Pre; paired *t*). (**e**,**f**) In animals pretreated with scopolamine at the dose (open triangle; 0.2 mg kg^−1^, i.p.) that completely blocked LFS-induced LTD (see Fig. 2a,b), application of LFS-300 30 min after i.c.v. injection of vehicle did not induce LTD, whereas application of LFS-300 30 min after i.c.v. injection of soluble Aβ_1–42_ induced a robust and stable LTD. As summarized in (**f**), at 3 h the EPSP measured 102.2±6.6% in the scopolamine+vehicle group (*n*=4, *P*>0.05 compared with Pre; paired *t*) and 71.2±5.8% in the scopolamine+Aβ group (*n*=6, *P*<0.05 compared with Pre or scopolamine+vehicle group; *t*-tests). Values are mean±s.e.m. Calibration: vertical, 2 mV; horizontal, 10 ms.

We wondered whether this Aβ-facilitated additional, mAChR-independent, LTD was due to the ability of Aβ to lower the threshold for LTD induction *in vivo.* We therefore used the weak LFS conditioning protocol (LFS-300). In addition to our standard soluble Aβ_1–42_ preparation we also tested a preparation of soluble Aβ_1–42_ enriched with protofibrils (Fig. 4). We combined the results obtained with the two synthetic Aβ_1–42_ preparations because there was no quantitative difference in their effects on LTD. The application of weak LFS-300 induced a large and robust LTD that was stable for at least 3 h in animals injected with Aβ_1–42_ (Fig. 5a,b), but not vehicle or a control, reverse sequence peptide Aβ_42–1_ (Fig. 5a,b). This dose (160 pmol) of Aβ_1–42_ did not affect baseline synaptic transmission (Fig. 5a,b) and consistent with a relatively selective action of Aβ on the mechanisms underlying LTD induction, the same dose applied after the LFS-300 conditioning stimulation failed to facilitate LTD (Fig. 5c,d). Moreover, the LTD induced by weak LFS-300 in the presence of Aβ, like the additional LTD induced by the strong LFS-900 protocol, was also mAChR-independent, not being blocked by scopolamine pretreatment (Fig. 5e,f).

**Figure 6:**
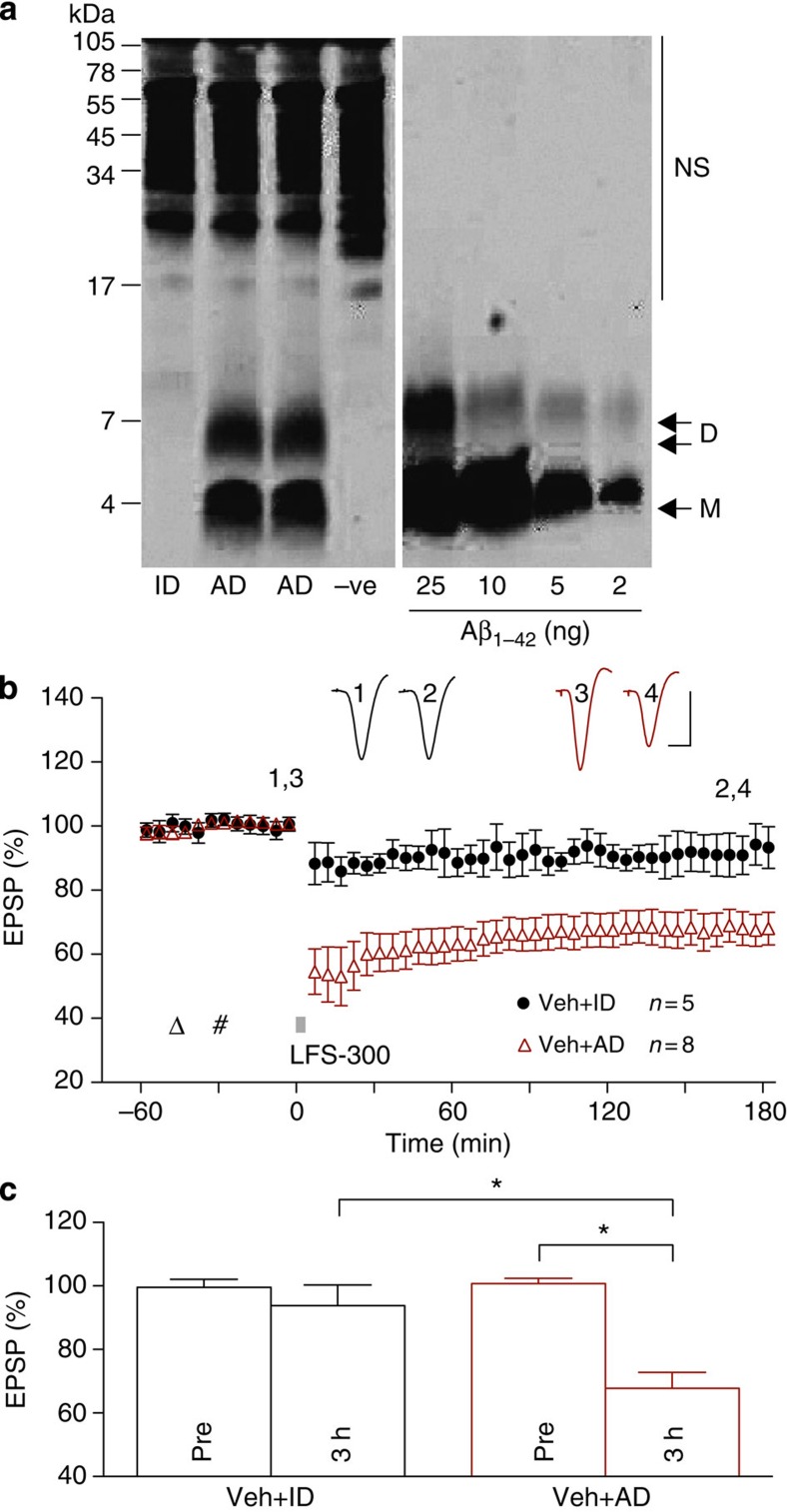
Aβ in AD brain TBS soluble extract facilitates LTD *in vivo.* (**a**) The TBS extract of AD2 was examined by immunoprecipitation/western blot as described in the Methods. The second and third lanes of the WB show duplicate samples of the buffer-exchanged AD2 extract that contained Aβ monomer (M) and SDS-stable Aβ dimers (D). The first lane shows that the immunodepleted sample had been effectively depleted of all detectable Aβ. Known amounts of synthetic Aβ_1–42_ were electrophoresed on the same gel to allow estimation of Aβ content in the test samples (~8.8 ng ml^−1^ and 5.6 ng ml^−1^ Aβ monomer and dimer, respectively). Molecular weight standards are indicated on the left and are given in kDa. Cross-reactive immunoglobulin-derived proteins that were detected when TBS buffer was immunoprecipitated are indicated (NS). (**b**,**c**) Similar to soluble synthetic Aβ_1–42_, acute i.c.v. injection of unmanipulated TBS extract of AD brain (AD, 5 μl) also enabled the induction of robust and persistent LTD by the weak LFS-300 protocol. In contrast, the same extract of AD brain that had been immunodepleted of Aβ using a polyclonal anti-Aβ antibody (ID) did not enable the induction of LTD by LFS-300. Triangle: Vehicle; hash: AD or ID. As summarized in (**c**) at 3 h the EPSP measured 67.8±4.8% in the AD group (*n*=8, *P*<0.05 compared with Pre; paired *t*) and 93.8±6.3% in the ID group (*n*=5, *P*>0.05 compared with Pre; *P*<0.05 compared with AD group; *t*-test). Values are mean±s.e.m. Calibration: vertical, 2 mV; horizontal, 10 ms.

Although synthetic Aβ is most commonly used in studies of synaptic plasticity disruption, it is important to determine whether similar effects are caused by natural Aβ. The presence of water-soluble SDS-stable Aβ dimer in post-mortem brain extracts is highly correlated with ante-mortem dementia status^31^ and such Aβ can inhibit LTP and promote LTD *in vitro*^5^,^8^. It is therefore of great interest to assess whether AD brain-derived Aβ can also facilitate LTD induction *in vivo*. Consequently, we tested the ability of Aβ in water-soluble extracts of two different AD brains to mimic the ability of synthetic Aβ_1–42_ to lower the threshold for LTD induction *in vivo*. As can be seen from the western blot of one of the AD brain extracts (Fig. 6a), Aβ runs on SDS gel predominantly as either monomer or dimer. These water-soluble SDS-stable species include a wide distribution of assemblies when analysed by size exclusion chromatography (SEC), ranging from monomer to ≥70 kDa (ref. 8). Similar to synthetic Aβ, the injection of Aβ-containing AD brain soluble extract enabled the induction of robust and stable LTD by LFS-300 (Fig. 6b,c). Importantly, immunodepletion of Aβ from the AD brain sample abrogated its ability to enable LTD induction. This finding indicates that soluble Aβ is responsible for the lowering of the LTD induction threshold by the AD brain extract. Which SDS-stable Aβ assembly is responsible for the facilitation of LTD by AD TBS brain extract remains to be determined.

### Aβ-facilitated LTD is NMDAR-independent

**Figure 7:**
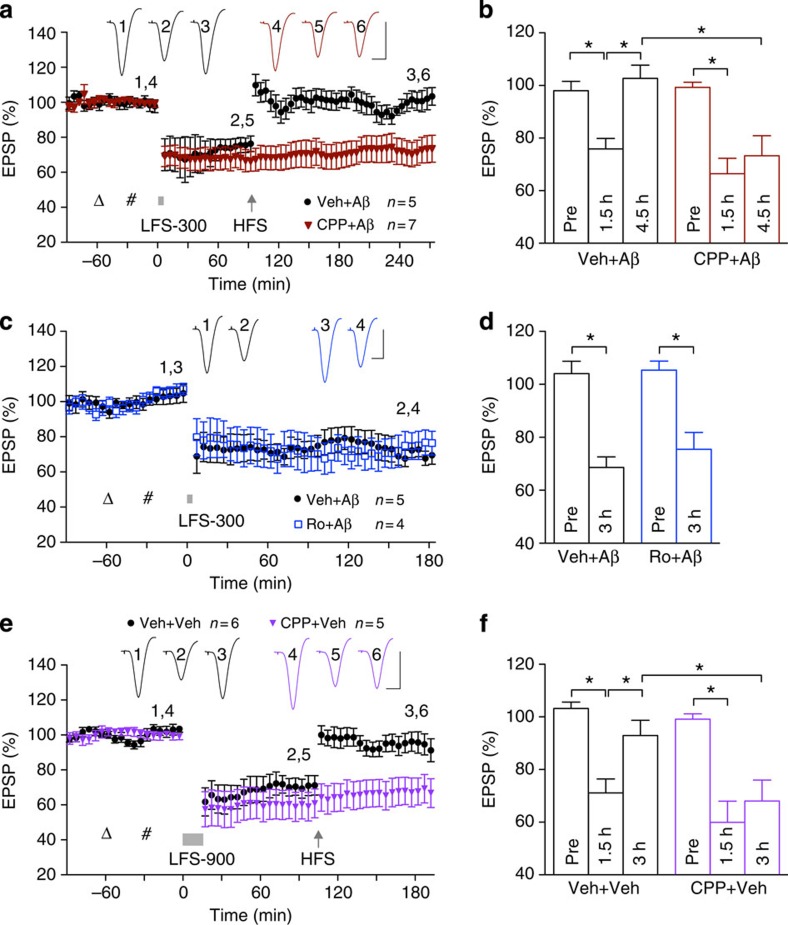
NMDAR antagonists do not affect LTD but prevent LTD reversal. (**a**,**b**) LFS-300 (bar) after Aβ_1–42_ (i.c.v., hash) triggered LTD that was reversed by HFS. The competitive antagonist CPP (open triangle; 10 mg kg^−1^, i.p.) did not affect Aβ-facilitated LTD but prevented de-depression. (**b**) Thus LTD at 1.5 h measured 75.9±4.0% (*n*=5) and 66.5±5.8% (*n*=7) in the vehicle+Aβ group and CPP+Aβ group, respectively (*P*<0.05 compared with Pre, one-way ANOVA-Tukey, *P*>0.05 between groups; two-way ANOVA followed by unpaired *t*). The EPSP measured 102.7±5.1% in the vehicle+Aβ group (at 3 h, *P*>0.05 compared with Pre and *P*<0.05 compared with 1.5 h post LFS) and 73.3±7.6% in the CPP+Aβ group (*P*>0.05 compared 1.5 h post LFS, *P*<0.05 compared with the vehicle+Aβ group). (**c**,**d**) Injection of Ro 25-6981 (open triangle; 6 mg kg^−1^, i.p.), a negative allosteric modulator of GluN2B-containing NMDARs, did not prevent Aβ_1–42_ (hash)-facilitated LTD (75.5±6.3% at 3 h, *n*=5, *P*<0.05 compared with Pre, *P*>0.05 compared with 68.6±4.0% in the vehicle+Aβ injection group; *t*-tests). (**e**,**f**) Control LTD, induced by LFS-900 (bar) was also reversed by HFS (arrow). CPP failed to significantly affect control LTD, but blocked de-depression. (**f**) Thus, LFS-900 induced LTD in controls (71.1±5.3% at 1.5 h, *n*=6, *P*<0.05 compared with Pre; one-way ANOVA-Tukey) and CPP-injected rats (59.9±8.0%, *n*=5, *P*<0.05 compared with Pre, *P*>0.05 compared with vehicle; two-way ANOVA followed by unpaired *t*). The EPSP measured 92.9±5.8% at 90 min in controls (*P*>0.05 compared with Pre) and 68.0±8.0% in the CPP group (*P*>0.05 compared with 1.5 h post LFS, *P*<0.05 compared with vehicle). Values are mean±s.e.m. Calibration: vertical, 2 mV; horizontal, 10 ms.

Because Aβ has been reported to promote NMDAR-dependent LTD^5^,^6^, we postulated that activation of NMDARs in the presence of Aβ may bypass the need for mAChRs in the induction of LTD *in vivo*. Contrary to our prediction, the NMDAR antagonist CPP, at a dose (10 mg kg^−1^, intraperitoneal (i.p.)) that completely blocks HFS-induced LTP^32^, did not affect the induction of LTD by LFS-300 in the presence of soluble Aβ_1–42_ (Fig. 7a,b). As CPP is a competitive antagonist and NMDARs containing GluN2B subunits are particularly implicated in Aβ-mediated synaptic plasticity disruption^12^,^13^,^14^,^15^, we also tested the GluN2B-selective negative allosteric modulator Ro 25-6981 (ref. 33)^33^. Using a dose (6 mg kg^−1^, i.p.) that prevents Aβ-mediated inhibition of LTP^12^, Ro 25–6981 had no effect on the facilitation of LTD by Aβ (Fig. 7c,d). We concluded that like control LTD induced by LFS-900 (Fig. 7e,f), Aβ-facilitated LTD induced by LFS-300 is NMDAR-independent.

### HFS-induced de-depression of LTD is NMDAR-dependent

In the light of the contrasting findings regarding the involvement of NMDARs in the disruptive effects of Aβ on LFS-induced LTD (present study) and HFS-induced LTP^12^,^13^,^14^,^15^, we also examined the effect of Aβ on another form of synaptic plasticity, HFS-induced de-depression. De-depression is the persistent reversal of LTD by conditioning stimulation and is believed to be an essential component of bidirectional synaptic plasticity^34^,^35^. Although the induction of control LTD did not require activation of NMDARs, the reversal of this LTD by HFS *in vivo* was NMDAR-dependent. Thus, whereas the NMDAR antagonist CPP did not affect control LTD induced by LFS-900, it completely prevented the reversal of this mAChR-dependent LTD by HFS conditioning stimulation (Fig. 7e,f). To our surprise, HFS-induced de-depression was not prevented by Aβ. Thus, HFS rapidly and persistently reversed Aβ-facilitated LTD (Fig. 7a,b). Moreover, HFS-induced de-depression of Aβ-facilitated LTD, like the persistent reversal of control LTD, was NMDAR-dependent, being abrogated in animals pretreated with CPP (Fig. 7a,b). This indicates that HFS-induced NMDAR-dependent de-depression is resistant to Aβ, unlike HFS-induced NMDAR-dependent LTP, as described previously^3^,^4^ and below. This lack of effect of Aβ on NMDAR-dependent de-depression, taken together with the inability of NMDAR antagonists to prevent the facilitation of LTD by Aβ, underlines the potential importance of non-NMDAR mechanisms in mediating the synaptic plasticity disrupting effects of Aβ *in vivo*.

### Aβ-facilitated LTD is mGlu5R-dependent

**Figure 8:**
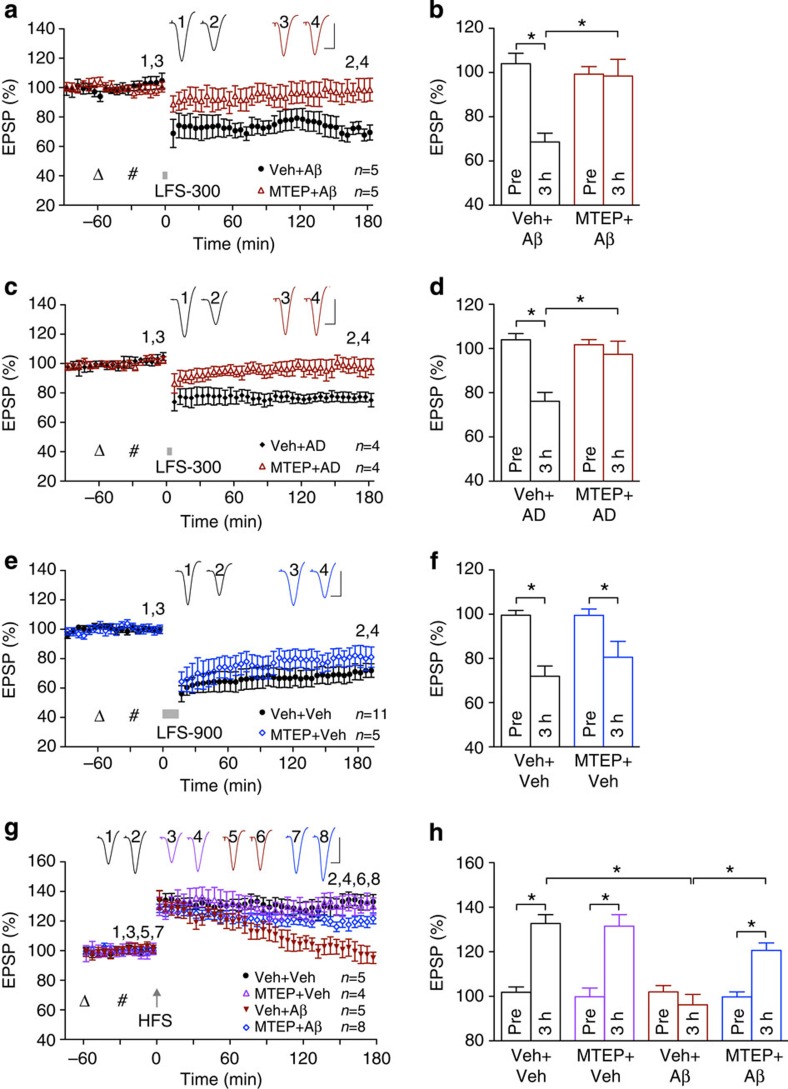
mGlu5R-dependence of Aβ-mediated disruption of both LTD and LTP but not control LTD or control LTP. (**a**,**b**) Systemic administration of the selective mGlu5R antagonist MTEP (open triangle; 3 mg kg^−1^, i.p.) completely prevented the induction of LTD by LFS-300 (bar) in animals injected i.c.v. with soluble Aβ_1–42_ (hash) (68.6±4.0% in the vehicle+Aβ group, *n*=5, *P*<0.05 compared with Pre, and compared with 98.4±7.6% in the MTEP+Aβ group, *n*=5, *P*>0.05 compared with Pre; *t*-tests). (**c**,**d**) Similarly, MTEP completely prevented the induction of LTD in animals injected with Aβ-containing AD brain extract. As summarized in (**d**) the EPSP measured 76.2±3.6% in the vehicle+AD group, *n*=4, (*P*<0.05 compared with Pre, and compared with 97.4 ±5.6% in the MTEP+AD group, *n*=4, *P*>0.05 compared with Pre; *t*-tests). (**e**,**f**) In contrast, the same dose of MTEP that prevented Aβ-facilitated LTD failed to significantly affect control LTD induced by LFS-900 (72.0±4.4% in the vehicle+vehicle group, *n*=11, *P*<0.05 compared with Pre, and *P*>0.05 compared with 80.6±7.2% in the MTEP+vehicle group, *n*=5, *P*<0.05 compared with Pre; *t*-tests). (**g**,**h**) i.c.v. injection of soluble Aβ_1–42_ (hash), at the dose that facilitated LTD, blocked LTP completely at 3 h post HFS. Although systemic administration of MTEP (3 mg kg^−1^) did not affect HFS-induced control LTP, it prevented Aβ-mediated impairment of LTP. As summarized in (**h**), HFS induced significant (*P*<0.05 compared with Pre; paired *t*) LTP in the vehicle control group (132.7±4.0%, *n*=5), MTEP+vehicle group (131.5±5.2%, *n*=4) and MTEP+Aβ group (120.6±3.4%, *n*=8), but not in the vehicle+Aβ group (96.2±4.7%, *n*=5), which was significantly different from the other groups (one-way ANOVA-Tukey). Values are mean±s.e.m. Calibration: vertical, 2 mV; horizontal, 10 ms.

Apart from NMDARs, metabotropic glutamate receptors, in particular the mGlu5R subtype, have been implicated in the synaptic plasticity disrupting actions of Aβ *in vitro*^5^,^8^,^36^. Bearing in mind the apparently differential roles of NMDARs in the effects of Aβ on different forms of synaptic plasticity, next we assessed the involvement of mGlu5R in both Aβ-mediated inhibition of LTP as well as Aβ-facilitated LTD *in vivo*. Remarkably, systemic administration of the selective mGlu5R antagonist (negative allosteric modulator) MTEP prevented both of these disruptive actions of Aβ without affecting either control LTP or control LTD. Thus, in animals administered with MTEP before intracerebroventricular (i.c.v.). injection of either synthetic or AD brain-derived Aβ the application of LFS-300 failed to induce LTD (Fig. 8a–d). Importantly, the same dose of MTEP had no effect on control LTD induced by LFS-900 (Fig. 8e,f), indicating that whereas Aβ-facilitated LTD is mGlu5R-dependent, this was not the case for the control mAChR-dependent LTD. Somewhat similarly, whereas Aβ_1–42_ strongly inhibited LTP in vehicle-pretreated animals, an identical HFS-triggered robust LTP in animals injected with MTEP followed by Aβ (Fig. 8g,h). These findings strongly indicate that Aβ enables LTD induction *in vivo* with an essential role of mGlu5, bypassing a requirement for activation of muscarinic ACh receptors. Moreover as MTEP prevented Aβ’s effects on both LTP and LTD, mGlu5Rs appear to be more pivotal to the synaptic plasticity disrupting actions of Aβ than NMDARs.

### Cellular prion protein mediates Aβ-facilitated LTD

**Figure 9:**
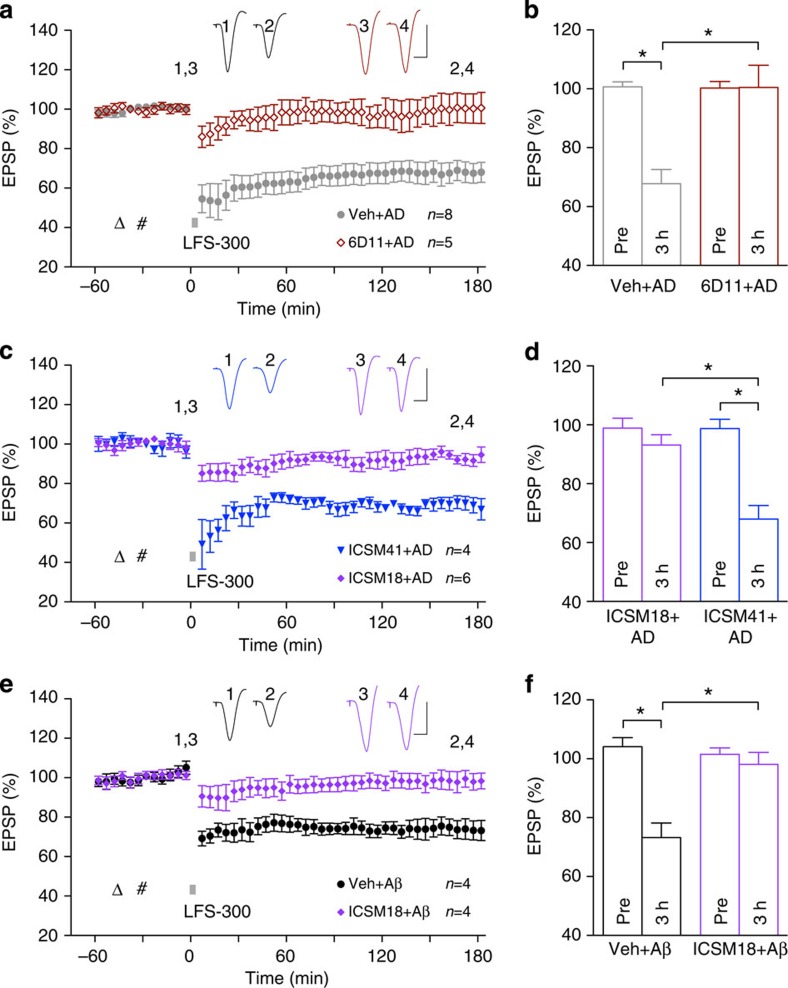
Cellular prion protein is necessary for the facilitation of LTD by Aβ. (**a**,**b**) Injection of 6D11 (triangle; 20 μg in 10 μl, i.c.v.), an antibody directed to the main binding site of Aβ on PrP^C^, 15 min before the injection of soluble Aβ-containing AD brain extract (hash) prevented the facilitation of LTD (interleaved experiments with vehicle+AD, from Fig. 5). As summarized in (**b**) at 3 h post LFS the EPSP measured 100.5±7.5%, *n*=5, in the 6D11+AD group (*P*>0.05 compared with Pre; *P*<0.05 compared with the vehicle+AD group; *t*-test). (**c**,**d**) Whereas ICSM18 (30 μg) prevented the facilitation of LTD by AD brain extract, the same dose of ICSM41 was ineffective. As summarized in (**d**) the EPSP measured 93.1±2.9%, *n*=6, in the ICSM18+AD group (*P*>0.05 compared with Pre and *P*<0.05 compared with 68.0±4.2%, *n*=4, in the ICSM41+AD group; *t*-test). (**e**,**f**) ICSM18 (3.75 μg) also prevented the facilitation of LTD by protofibril Aβ_1–42_. As summarized in (**f**) the EPSP measured 98.1±3.7% at 3 h post LFS in the ICSM18+Aβ_1–42_ group (*P*>0.05 compared with Pre, *n*=4; and *P*<0.05 compared with 73.2±4.5%, *n*=4, in the vehicle+Aβ_1–42_ group; *t*-test). Values are mean±s.e.m. Insets show representative EPSP traces at the times indicated. Calibration: vertical, 2 mV; horizontal, 10 ms.

**Figure 10:**
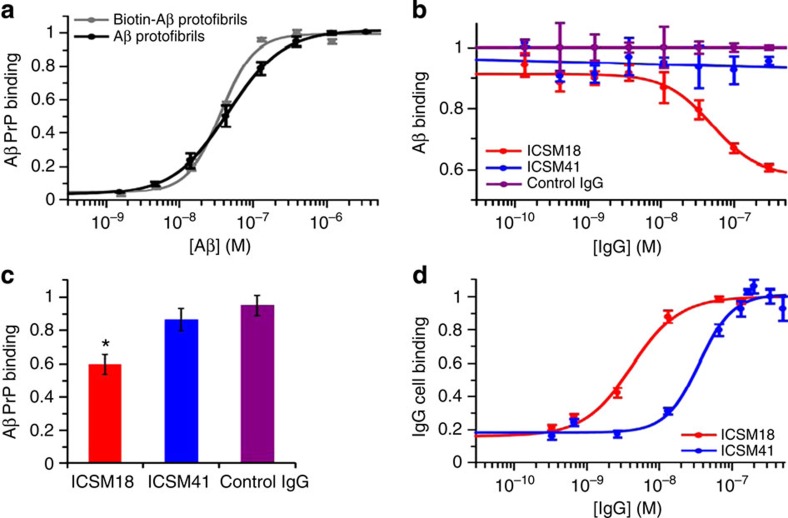
Characterization of the interactions between Aβ and PrP^C^ and anti-PrP^C^ antibodies. (**a**) Both Aβ_1–42_ and biotinylated Aβ_1–42_ protofibrils bind recombinant PrP^C^ at low nanomolar concentrations (*n*=3±s.e.m.). (**b**,**c**) Unlike ICSM18, ICSM41 did not prevent Aβ_1–42_ protofibril binding to PrP^C^ (**b**, *n*=3; **c**, *n*=9, mean±s.e.m.). *P<0.05 compared to control IgG (BRIC222), Kruskal–Wallis one-way ANOVA with Dunn’s multiple comparison test. (**d**) FACS analysis revealed that ICSM18 bound to N2A cells, which express glycosylated mature PrP^C^, with an approximately eightfold higher affinity than ICSM41 (XC_50_ 4±1 nM and 33±7 nM, respectively, *n*=4, mean±s.e.m.).

The question arises as to whether or not the facilitation of LTD by Aβ shares other common mechanisms with LTP inhibition by Aβ. Aβ oligomers can bind very potently and selectively to cellular prion protein especially in a region that encompassed the amino-acid sequence 95–105, and thereby mediate inhibition of LTP by synthetic Aβ_1–42_ (ref. 25). The disease relevance of this finding is underscored by the PrP^C^-dependence of the inhibition of LTP by Aβ oligomer-containing soluble extract of AD brain^37^. We examined the role of PrP^C^ in mediating the facilitation of LTD by AD brain Aβ and synthetic Aβ_1–42_ using monoclonal antibodies to PrP^C^. We started with the previously characterized anti-PrP^C^ antibody 6D11, with an epitope that falls within the amino-acid 93–109 sequence, thereby preventing Aβ_1–42_ oligomer binding and inhibition of LTP^25^. Pretreatment with 6D11 completely prevented the facilitation of LTD by Aβ-containing soluble AD brain extract (Fig. 9a,b). In order to further explore the role of PrP^C^, we compared the effect of two other high-affinity anti- PrP^C^ antibodies (Fig. 9c,d). ICSM18, an antibody directed to helix-1 of PrP^C^, is known to inhibit Aβ binding to PrP^C^ and to prevent the LTP disrupting effect of AD brain extracts^37^. ICSM41 is an antibody to the structured region of PrP^C^ with an undefined epitope that does not map to the Aβ-binding region^38^,^39^. Although ICSM41 binds with similar high affinity to recombinant PrP^C^ as ICSM18 (IC_50_: 0.41±0.04 and 0.3±0.1 nM, respectively, *n*=9, mean±s.e.m.), unlike ICSM18, ICSM41 did not prevent Aβ_1–42_ protofibril binding to PrP^C^ (Fig. 10b,c). Consistent with the differential ability of these two antibodies to prevent Aβ_1–42_ binding to PrP^C^, ICSM18 abrogated the facilitation of LTD by soluble AD brain extract, whereas the same dose of ICSM41 had no effect (Fig. 9c,d). These findings provide strong evidence that PrP^C^ is required for the enablement of LTD by the most disease relevant form of soluble Aβ, Aβ from AD brain. We also tested the ability of ICSM18 to prevent the facilitation of LTD by synthetic Aβ_1–42_. Aβ from water-soluble extracts of AD brain contain a mixture of high- and low-molecular weight components^8^, some of which bind to PrP^C^ with high affinity^40^,^41^. In the case of synthetic Aβ, protofibrillar assemblies bind most avidly to PrP^C^ (ref. 26) (see also Fig. 10a). We tested an eightfold lower dose of ICSM18 in this study because we found that ICSM18 bound to N2A cells, which express glycosylated mature PrP^C^, with an approximately eightfold higher affinity than ICSM41 (XC_50_ 4±1 and 33±7 nM, respectively) (Fig. 10d). We found that this dose of ICSM18 completely abrogated the facilitation of LTD by protofibril Aβ_1–42_ (Fig. 9e,f). On the basis of the present and our previous^37^ findings, PrP^C^ appears to be a key site of binding and action for Aβ-mediated disruption of both NMDAR-dependent and independent synaptic plasticity *in vivo*.

## Discussion

We describe here for the first time the *in vivo* induction by synaptic stimulation of an mAChR-dependent homosynaptic LTD. The induction of mAChR-dependent LTD does not require NMDA or mGlu5R activation. Moreover, both chemically synthesized and human brain-derived Aβ enhanced synaptically induced LTD *in vivo*. Remarkably, in Aβ-treated animals the additional LTD does not require mAChRs, leaving mAChR-dependent LTD relatively intact. However, like mAChR-dependent LTD, the Aβ-facilitated LTD is NMDAR-independent. We found evidence that mGlu5R activation usurps the requirement for mAChRs to enable LTD induction via a process dependent on PrP^C^. Furthermore, Aβ-mediated inhibition of LTP also requires mGlu5R and PrP^C^, placing Aβ–PrP^C^–mGlu5R interactions central to the synaptic plasticity disrupting actions of Aβ *in vivo*.

LTD that requires mAChR activation has been proposed to be essential for certain forms of learning^21^, and the preservation of mAChR-dependent hippocampal LTD as animals age may be critical for maintaining cognitive performance^22^. The apparent dearth of studies of mAChR-dependent LTD *in vivo* may be owing to difficulties in optimizing suitable synaptic stimulation protocols. The present approach utilizes the insights gained from investigations of mAChR-dependent LTD in slices of cerebral cortex^28^. Currently used *in vitro* synaptic stimulation protocols to induce mAChR-dependent LTD at CA3-to-CA1 synapses have been reported to induce an LTD that is at least partly inhibited by mAChR antagonists^19^. The present finding that synaptic conditioning stimulation can induce LTD that is completely blocked by scopolamine provides strong evidence that mAChR-dependent LTD that lasts for over 5 h can be induced by endogenously released ACh *in vivo* and therefore supports its proposed role in synaptic information storage.

Because previous reports had indicated that *in vitro*, Aβ strongly impairs mAChR-mediated signalling^29^ that may underlie mAChR-dependent LTD in the cerebral cortex^28^, we predicted that mAChR-dependent LTD in the hippocampus would be inhibited by Aβ *in vivo*. To our surprise, Aβ enabled additional LTD while at the same time leaving a scopolamine sensitive component of LTD relatively unscathed. It was apparent that Aβ usurped mAChR-dependent LTD by lowering the synaptic stimulation threshold to induce another form of LTD that was mAChR-independent. The mechanisms of the additional LTD, however, appear to be at least partly shared with mAChR-dependent LTD, as the initial phase of the control LTD was partly occluded by the Aβ-enabled LTD.

Particularly surprising was the apparent lack of involvement of NMDARs in the facilitation of LTD by Aβ, especially in view of the presumed essential role of NMDARs in the relatively selective binding of Aβ oligomers to synapses^42^. Moreover, antagonists of GluN2B subunits prevent Aβ-mediated facilitation of NMDAR-dependent LTD^5^,^6^ and inhibition of NMDAR-dependent LTP^12^,^13^,^14^,^15^. These findings have led to the elucidation of a key role of GluN2B subunits in mediating the synaptic plasticity disrupting actions of Aβ and have been extended to include many other deleterious effects of Aβ^40^,^43^. However, based on the present results, targeting GluN2B is unlikely to prove to be an effective therapeutic strategy on its own and underlines the need to also explore non-NMDAR mechanisms.

Further undermining the putative primacy of NMDARs in the synaptic actions of Aβ was the finding that Aβ did not significantly affect NMDAR-dependent de-depression. This is all the more remarkable considering that Aβ strongly inhibited NMDAR-dependent LTP at these same synapses using the same HFS induction protocol. Previous research has found that the persistent reversal of LTD by conditioning stimulation requires the recruitment of different signalling pathways to those usually necessary for LTP induction^44^,^45^. Thus the lack of inhibition of NMDAR-dependent de-depression at these synapses indicates that the inhibition of LTP by Aβ is not due to the dependence of LTP on NMDARs. Furthermore, the present findings indicate that pharmacological inhibition of NMDARs may prevent potentially physiological reversal of LTD and leave any deleterious effects of Aβ-facilitated NMDAR-independent LTD unopposed.

The present findings underscore a much more central role for the mGlu5R in mediating the synaptic plasticity disrupting effects of Aβ and suggest that the lowering of the threshold for LTD and inhibition of LTP are two sides of one coin. Our finding that Aβ-facilitated LTD, like Aβ-mediated inhibition of LTP, is blocked by antibodies that prevent Aβ binding to PrP^C^ provides an explanation for the pivotal role of mGlu5Rs. Previous research^46^ has revealed that Aβ acts as an extracellular scaffold to promote the inappropriate synaptic mobilization and activation of mGlu5R on cultured neurons. The membrane binding of Aβ is prevented by both anti-mGlu5R and anti-PrP^C^ antibodies in a non-additive manner^46^, consistent with the key role of PrP^C^ in the binding of the Aβ oligomer to plasmalemma^25^. The aberrant clustering of mGlu5R at synapses by Aβ by binding to PrP^C^ may trigger disruptive signalling activity that can enable LTD and inhibit LTP induction. Very recently direct evidence that PrP^C^ mediates multiple effects of Aβ oligomers, including dendritic spine loss in cultured neurons, by a direct physical linkage of PrP^C^ with mGlu5Rs at or near the postsynaptic membrane was reported^27^. If the formation of Aβ–PrP^C^–mGlu5R complexes is primary, then the requirement for NMDARs that contain GluN2B subunits in the inhibition of LTP by Aβ is likely to be a downstream consequence. Indeed, mGlu5Rs provide a transduction link in the Aβ–PrP^C^ complex-mediated transmembrane coupling to NR2B subunits via activation of the tyrosine kinase Fyn^27^,^40^,^47^. In addition to Fyn, an Aβ–PrP^C^–mGlu5R-mediated dysregulation of intracellular Ca^2+^, eukaryotic elongation factor 2 and Arc^27^ may contribute to synaptic plasticity disruption^11^ by Aβ *in vivo*.

Overall, the present research provides strong evidence that an Aβ–PrP^C^–mGlu5R triad is critical for synaptic plasticity disruption, enabling an NMDAR-independent LTD to usurp mAChR-dependent LTD and inhibit NMDAR-dependent LTP. Selectively targeting this Aβ–PrP^C^–mGlu5R triad offers many possible means of preventing dysfunction of critical brain plasticity mechanisms in early AD.

## Methods

### Animals and surgery

Adult (250–350 g, 8–11 weeks old) male Wistar rats (BioResources Unit, Trinity College, Dublin) were used in all experiments. The animals were housed under a 12-h light-dark cycle at room temperature (19–22 °C). Before the surgery, animals were anesthetized with urethane (1.5–1.6 g kg^−1^, i.p.). Lignocaine (10 mg, 1% adrenaline, subcutaneously) was injected over the area of the skull, where electrodes and screws were to be implanted. The body temperature of the rats was maintained at 37–38 °C with a feedback-controlled heating blanket. The animal care and experimental protocol were approved by the Department of Health, Republic of Ireland.

### Cannula implantation

In order to inject drugs or Aβ into the brain, a stainless-steel cannula (22 gauge, 0.7 mm outer diameter) was implanted above the right lateral ventricle (1 mm lateral to the midline and 4 mm below the surface of the dura). i.c.v. injection was made via an internal cannula (28 gauge, 0.36 mm outer diameter). The solutions were injected in a 5 μl volume over a 3-min period or 10 μl volume over a 6-min period. Verification of the placement of cannula was performed post mortem by checking the spread of ink dye after i.c.v. injection.

### Electrode implantation

Monopolar recording electrodes were constructed from Teflon-coated tungsten wires (75 μm inner core diameter, 112 μm external diameter) and twisted bipolar stimulating electrodes were constructed from Teflon-coated tungsten wires (50 μm inner core diameter, 75 μm external diameter) separately^12^. Field excitatory postsynaptic potentials (EPSPs) were recorded from the stratum radiatum in the CA1 area of the right hippocampus in response to stimulation of the ipsilateral Schaffer collateral-commissural pathway. Electrode implantation sites were identified using stereotaxic coordinates relative to bregma, with the recording site located 3.4 mm posterior to bregma and 2.5 mm lateral to midline, and stimulating site 4.2 mm posterior to bregma and 3.8 mm lateral to midline. In some animals, another stimulating electrode was implanted at a site located 2.5 mm posterior to bregma and 2.2 mm lateral to the midline. The final placement of electrodes was optimized by using electrophysiological criteria and confirmed via post-mortem analysis.

### Electrophysiology

Test EPSPs were evoked by a single square-wave pulse (0.2 ms duration) at a frequency of 0.033 Hz and an intensity that triggered a 50% maximum EPSP response. LTD was induced using 1 Hz LFS consisting of 900 pulses (0.2 ms duration). During the LFS the intensity was raised to trigger EPSPs of 95% maximum amplitude. A relatively weak LFS protocol, used to study the Aβ-mediated facilitation of LTD, consisted of 300 pulses (0.2 ms duration) at 1 Hz, with an intensity that evoked 95% maximum amplitude. LTP was induced using 200 Hz HFS consisting of one set of 10 trains of 20 pulses (inter-train interval of 2 s). The stimulation intensity was raised to trigger EPSPs of 75% maximum during the HFS. None of the conditioning stimulation protocols elicited any detectible abnormal changes in background EEG, which was recorded from the hippocampus throughout the experiments.

### Compounds and antibodies

Scopolamine (Sigma), mecamylamine (Sigma), (R,S)-3-(2-carboxypiperazin-4-yl)propyl-1-phosphonic acid (CPP, Ascent Scientific, Weston-Super-Mare, UK) and 3-((2-methyl-1,3-thiazol-4-yl)ethynyl)pyridine hydrochloride (MTEP hydrochloride, Ascent Scientific) were prepared in distilled water and diluted with saline to the required concentration. Pirenzepine (Ascent Scientific) was prepared in distilled water. (αR,βS)-α-(4-hydroxyphenyl)-β-methyl-4-(phenylmethyl)-1-piperidinepropanol hydrochloride (Ro 25–6981, Sigma) was dissolved in DMSO (dimethylsulphoxide) and diluted in saline. The following monoclonal antibodies, prepared in phosphate-buffered saline (PBS), were used in this study: 6D11 (Covance, # SIG-39810); ICSM18, ICSM41 and BRIC222 (D-Gen, UK, # ICSM18, ICSM41 and BRIC222)).

### Synthetic Aβ

We made two main different preparations of synthetic Aβ, soluble and protofibril Aβ_1–42_. Our standard, soluble Aβ_1–42_ (Bachem or Biopolymer Laboratory, UCLA Medical School) was prepared as a stock solution of 64 μM in mild alkali (0.1% ammonium hydroxide) in milliQ water (Millipore Corporation, Ireland) to avoid isoelectric precipitation and then centrifuged at 100,000 *g* for 3 h to remove any fibril aggregates. An aliquot of the supernatant was taken to estimate peptide concentration using the micro BCA protein assay (Thermo-Fisher Scientific Life Science Research Products, Rockford, IL) and the remaining supernatant was stored at −80 °C until required. Whereas the test dose (160 pmol) of soluble Aβ_1–42_ did not affect baseline transmission in the absence of LFS (see Results), double this dose (320 pmol, i.c.v.) caused a small (~15%) decrease in baseline at 3 h.

Differentially aggregated protofibril Aβ_1–42_ and biotinylated Aβ_1–42_ were synthesized, and purified by Dr James I. Elliott at Yale University (New Haven, CT). Peptide (~10–20 mg) was weighed into a screw-cap 50-ml Sterilin tube, dissolved in anhydrous DMSO with gentle mixing for 2 min to produce a 5-mM solution and then diluted to 100 μM in phenol red-free Ham’s F12 medium (Caisson Labs) and vortexed for 15 s. Samples were aggregated without shaking for 48 h, transferred to a 2-ml eppendorf tube, centrifuged at 16,100 *g* for 20 min to remove any large preformed aggregates and the upper 90% for each solution collected, aliquoted, snap frozen in liquid N_2_ and stored at −80 °C. Samples were then tested for the presence of large protofibrillar assemblies, known to bind avidly to PrP and cause PrP-dependent toxicity^26^. Aβ_1–42_ protofibrils used for electrophysiology were further dialysed against 2 × 5 l of PBS in an 8000 MWCO semi-permeable membrane to ensure all DMSO and cell media were exchanged before freezing and characterization.

### Electron microscopy

Five microlitre of peptide solution was applied to glow-discharged carbon-coated copper grids and left to bind for 60 s. Excess solution was removed using grade 4 Whatman filter paper. Samples were negatively stained with 2% uranyl acetate for 30 s, blotted then allowed to air dry. Images were acquired on an FEI Tecnai T10 electron microscope operating at 100 kV and recorded on a 1k × 1k charged couple device camera (Gatan) at a typical magnification of 34,000 with a pixel size of 5.03 Å.

### SEC and multi-angle light scattering

Aliquots (0.33 ml) of Aβ_1–42_ protofibrils were injected onto a Superdex 200 10/30 column (GE Healthcare) and eluted with PBS at a flow rate of 0.5 ml min^−1^ using an Agilent HPLC and peptide elution monitored by absorbance at 275 nm. Light scattering was performed using a Wyatt DAWN HELEOS II multi-angle light scattering module with Aβ concentrations calculated using the refractive index.

### TBS extract of human brain

AD brain 1 was obtained and used in accordance with the UCD Human Research Ethics Committee guidelines (under approval LS-E-10-10-Walsh). AD brain 2 was obtained and used in accordance with the Partner's Institutional Review Board (Walsh BWH 2011). In both cases informed consent was obtained from subjects. Samples of temporal cortex were obtained from 2 AD cases referred to as AD1 and AD2. AD1 was from an 85-year-old male with dementia and fulminant amyloid and tangle pathology (Braak stage=4) and was provided by Drs Dykoski and Cleary of Minneapolis VA Health Care System, and potently inhibits LTP^48^. AD2 was from an 81-year-old female who died with severe AD and was kindly provided by Dr Cindy Lemere of Brigham and Women’s Hospital. Frozen cortex (0.9 g) was allowed to thaw on ice, chopped into small pieces and homogenized in 4.5 ml of ice-cold 20 mM Tris–HCl, pH 7.4, containing 150 mM NaCl with 25 strokes of a Dounce homogenizer (Fisher, Ottawa, Ontario, Canada)^31^,^48^. Water-soluble Aβ was separated from membrane-bound and plaque Aβ by centrifugation at 91,000 *g* and 4 °C in a TLA 55 rotor (Beckman Coulter, Fullerton, CA, USA) for 78 min. To eliminate bioactive small molecules the supernatant was exchanged into ammonium acetate. Thereafter, extracts were divided into two parts: one aliquot was immunodepleted of Aβ by three rounds of 12-h incubations with our anti-Aβ antibody, AW8 (ref. 31)^31^, and protein A at 4 °C. The second portion was not manipulated in any way and is simply referred to as AD. Aliquots of samples were stored at −80 °C or used to assess Aβ content with a sensitive immunoprecipitation/western blot procedure. Our rabbit polyclonal antibody, AW8, was used (at a dilution of 1:80) for immunoprecipitation and a combination of the anti-Aβ40 and Aβ42 monoclonal antibodies, 2G3 and 21F12 (each at 1 μg ml^−1^) for western blot. Aβ concentration was estimated by reference to known quantities of synthetic Aβ_1–42_. Antibodies 2G3 and 21F12 were kindly provided by Drs P. Seubert and D. Schenk (Elan Pharmaceuticals).

### Aβ-binding DELFIA

Aβ binding to our recombinant PrP^C^ (refs 26, 37) was determined by an enzyme-linked immunosorbent assay (ELISA)-based protocol detected by the dissociation-enhanced lanthanide fluorescent immunoassay (DELFIA). Fifty microlitres of 1 μM human huPrP_23–231_ (10 mM sodium carbonate, pH 9.6) was bound to medium binding 96-well white plates (Greiner) with shaking at 400 r.p.m. for 1 h at 37 °C, washed with 3 × 300 μl of PBS (0.05% Tween-20), blocked with 300 μl Superblock (Thermo Scientific) with shaking at 400 r.p.m. at 37 °C for 1 h and washed with 3 × 300 μl of PBS (0.05% Tween-20). Fifty microlitres of Aβ_1–42_ protofibrils were incubated in PBS (0.05% Tween-20, 0.1% BSA) for 1 h at 25 °C with shaking at 400 r.p.m. and washed with 3 × 300 μl of PBS (0.1% Tween-20). Aβ was detected using 50 μl of 1 μg ml^−1^ 6E10 (Covance, # SIG-39320) in DELFIA assay buffer (PerkinElmer) for 1 h at 25 °C with shaking at 400 r.p.m., washed with 3 × 300 μl of PBS (0.05% Tween-20) and incubated for 1 h at 25 °C with shaking at 400 r.p.m. with 300 ng ml^−1^ of DELFIA Eu-N1 anti-mouse antibody in DELFIA assay buffer (PerkinElmer, # 4002-0010), washed with 3 × 300 μl of PBS (0.05% Tween-20) before enhancing with 100 μl of DELFIA Enhancement Solution (PerkinElmer)^49^. Biotinylated Aβ_1–42_ protofibrils were detected using a 1:2,000 dilution of DELFIA Eu-N1 streptavidin (PerkinElmer, # 1244-360), washed with 3 × 300 μl of PBS (0.05% Tween-20) before enhancing with 100 μl of DELFIA Enhancement Solution (PerkinElmer, # 4001-0010). Plates were scanned for time-resolved fluorescence intensity of the europium probe (*λ*_ex_=320 nm, *λ*_em_=615 nm) using a PerkinElmer EnVision plate reader. Apparent XC_50_ values were calculated using a four-parameter XC_50_ curve with the maximum plateau signal for a given series used to define full occupancy.

### Anti-PrP antibody binding DELFIA

Fifty microlitres of 150 nM of our huPrP_23–231_^26^,^37^ (10 mM sodium carbonate, pH 9.6) was bound to high-binding 96-well white plates (Greiner) with shaking at 400 r.p.m. for 1 h at 37 °C, washed with 3 × 300 μl of PBS (0.05% Tween-20), blocked with 300 μl Superblock (Thermo Scientific) with shaking at 400 r.p.m. at 37 °C for 1 h and washed with 3 × 300 μl of PBS (0.05% Tween-20). Fifty microlitres of ICSM18 or ICSM41 (concentration-response curve, D-Gen, # ICSM18 and ICSM41) were incubated in DELFIA assay buffer (PerkinElmer) for 1 h at 25 °C with shaking at 400 r.p.m. and washed with 3 × 300 μl of PBS (0.1% Tween-20). ICSM antibodies were detected by 50 μl of 100 ng ml^−1^ of DELFIA Eu-N1 anti-mouse antibody (PerkinElmer, # AD0207) in DELFIA assay buffer (PerkinElmer), washed with 3 × 300 μl of PBS (0.05% Tween-20) before enhancing with 100 μl of DELFIA Enhancement Solution (PerkinElmer)^49^.

### Anti-PrP antibody-mediated Aβ-inhibition DELFIA

Fifty microlitres of 1 μM human huPrP_23–231_ (10 mM sodium carbonate, pH 9.6) was bound to medium-binding 96-well white plates (Greiner) with shaking at 400 r.p.m. for 1 h at 37 °C, washed with 3 × 300 μl of PBS (0.05% Tween-20), blocked with 300 μl Superblock (Thermo Scientific) with shaking at 400 r.p.m. at 37 °C for 1 h and washed with 3 × 300 μl of PBS (0.05% Tween-20). Fifty microlitres of ICSM18 or ICSM41 (1 μg ml^−1^) were incubated in PBS (0.05% Tween-20, 0.1% BSA) for 1 h at 25 °C with shaking at 400 r.p.m. and washed with 3 × 300 μl of PBS (0.1% Tween-20). Biotinylated Aβ_1–42_ protofibrils were incubated in PBS (0.05% Tween-20, 0.1% BSA) for 30 min at 25 °C with shaking at 400 r.p.m. and washed with 3 × 300 μl of PBS (0.1% Tween-20). Aβ was detected using a 1:2,000 dilution of DELFIA Eu-N1 streptavidin (PerkinElmer), washed with 3 × 300 μl of PBS (0.05% Tween-20) before enhancing with 100 μl of DELFIA Enhancement Solution (PerkinElmer).

### FACS

N2a cells (mouse neuroblastoma, ATCC) were harvested and washed with PBS, blocked with FcγRIIb/CD16-2 (1 μg ml^−1^, #18867, Santa Cruz) for 30 min at 4 °C. Cells were incubated with different concentrations of ICSM18 or ICSM41 antibodies (concentration response from 0.05–75 μg ml^−1^) for 45 min at 4 °C. Subsequently, rinsed cells were stained with Alexa488- or FITC-conjugated anti-mouse antibodies (2 μg ml^−1^, # A-11001 and # F 2761, respectively, Invitrogen), fixed in 2% PFA for 10 min at 22 °C, stained with DAPI and kept at 4 °C until analysis. Samples were analysed on a CyAn ADP High Performance Flow Cytometer equipped with a 488 nm argon laser.

### Data analysis

The magnitude of LTD is expressed as the percentage of pre-LFS baseline EPSP amplitude (±s.e.m.). The sample size was chosen based on our knowledge of what is appropriate for *in vivo* electrophysiology to determine whether synaptic plasticity is induced or affected by Aβ or other interventions^3^,^6^,^10^,^12^. No data were excluded, and control experiments were interleaved randomly throughout. Two-tailed paired Student’s *t*-tests (paired *t*) or one-way ANOVA with Tukey’s multiple comparison test (one-way ANOVA-Tukey) were used to evaluate LTD within groups, and two-way ANOVAor unpaired Student’s *t*-tests (unpaired *t*) were used to compare between groups. Kruskal–Wallis one-way ANOVA with Dunn’s multiple comparison test was used to compare the effects of antibodies on Aβ binding to recombinant PrP^C^. A *P*<0.05 was considered as statistically significant.

## Author contributions

N.-W.H. and M.J.R. conceived the study; N.-W.H., A.J.N., D.M.W. and M.J.R. designed the research; N.-W.H. and D.Z. performed and analysed electrophysiology experiments; A.J.N. prepared the differentially aggregated Aβ and characterized it and the ICSM antibodies A.J.M. isolated and characterized human brain extracts; T.O’M. characterized the standard soluble Aβ_1–42_ preparation; S.A.P. performed FACS experiments; C.T. performed EM analysis; N.-W.H., A.J.N., J.C., D.M.W. and M.J.R. wrote the paper.

## Additional information

**How to cite this article:** Hu, N.-W. *et al.* mGlu5 receptors and cellular prion protein mediate amyloid-β-facilitated synaptic long-term depression *in vivo*. *Nat. Commun.* 5:3374 doi: 10.1038/ncomms4374 (2014).
